# ACTIVITY RECORDING CAPILLARY FEEDER (ARC): A HIGH-THROUGHPUT BEHAVIORAL PHENOMIC ASSESSMENT IN DROSOPHILA

**DOI:** 10.1093/geroni/igae098.3188

**Published:** 2024-12-31

**Authors:** Serena Wu, Vivian Pham, Tiffany Nguyen, Mahtab Jafari

**Affiliations:** University of California Irvine, Irvine, California, United States; University of California Irvine, Irvine, California, United States; University of California Irvine, Irvine, California, United States; University of California Irvine, Irvine, California, United States

## Abstract

Drosophila melanogaster is not only a well-established model organism in aging research but it is also considered an emerging animal model to screen and evaluate potential anti-aging compounds. Our lab developed an algorithm to screen and identify natural products and botanical extracts with lifespan extension and healthspan-improving properties. Once a compound or extract was shown to increase lifespan, we performed various phenotypic assays to determine its impact on biomarkers of health in Drosophila. This work established Drosophila as a model system in pre-clinical aging research. However, traditional behavioral phenotypic assays such as geotaxis are laborious and time-consuming, hampering screening efficiency. To address this challenge in the emerging field of phenomics, we have utilized an automated system, originally developed by Ja Lab, University of Florida, called the Activity Recording Capillary feeder assay (ARC). This recording system leverages high-resolution machine vision to monitor Drosophila behaviors such as movement, sleep, and feeding dynamics. This innovative approach enables rapid and simultaneous assessment of multiple behavioral parameters, offering greater reproducibility and scalability than conventional methods. In this work, we present preliminary data on the feasibility of using ARC as a high-throughput phenomics screening modality to evaluate natural product induced changes in aging-related phenotypes such as locomotion. This work was supported by UCI Calit2.

